# A flexible piezoelectric sensor for non-contact monitoring of respiratory and heart rates: accuracy and clinical feasibility under simulated dental treatment conditions

**DOI:** 10.1007/s00784-026-07161-7

**Published:** 2026-09-26

**Authors:** Fumi Kozu, Maho Suyama, Hiroyuki Kurakami, Chiho Kudo, Hiroshi Hanamoto

**Affiliations:** 1 Department of Dental Anesthesiology, Graduate School of Dentistry, The University of Osaka, Suita, Japan; 2 Department of Dental Anesthesiology, Graduate School of Biomedical and Health Sciences, Hiroshima University, 1-2-3, Kasumi, Minami-ku, Hiroshima, Japan; 3 Institute for Clinical and Translational Science, Nara Medical University Hospital, Kashihara, Nara Japan

**Keywords:** Dental chair, Piezoelectric sensor, Respiratory rate, Heart rate, Non-contact monitoring, Dental treatment

## Abstract

**Objectives:**

Vital signs are not routinely monitored in dental practice, potentially delaying the recognition of medical emergencies. This study aimed to evaluate the accuracy of a piezoelectric sensor (Taido Sensor, Sumitomo Riko Co., Ltd., Nagoya, Japan) for measuring respiratory rate (RR) and heart rate (HR). Resilience against vibrations from cutting instruments was also assessed.

**Materials and methods:**

Fifteen healthy participants were monitored on a dental chair fitted with the sensor. Respiratory inductive plethysmography and electrocardiography served as reference methods. Three protocols were executed: (1) controlled breathing at 6–28 breaths/min; (2) HR recovery following a 3-min Master’s two-step exercise test; and (3) clinical vibration simulated using an ultrasonic scaler, air turbine, and micromotors. RR and HR were derived from 30-s and 10-s waveform segments, respectively. Agreement was assessed using Lin’s concordance correlation coefficient (CCC) and Bland–Altman analysis.

**Results:**

The CCC for RR during controlled breathing was 0.992, and that for HR post-exercise was 0.982. CCCs remained excellent for both RR (0.968–0.997) and HR (0.954–0.997) under various dental vibrations. Bland–Altman analysis revealed minimal fixed bias and clinically acceptable limits of agreement across all conditions.

**Conclusions:**

The Taido Sensor demonstrates high concordance with reference methods for vital signs measurements in routine dental practice, maintaining accuracy despite mechanical interference from dental instruments.

**Clinical relevance:**

Owing to its contactless design and ability to provide reliable data from short segments, the Taido Sensor could be a valuable tool for monitoring real-time vital signs during routine dental procedures, thereby enhancing patient safety.

**Supplementary Information:**

The online version contains supplementary material available at https://doi.org/10.1007/s00784-026-07161-7.

## Introduction

Complications during dental procedures are often accompanied by physiological changes in vital signs, such as an increased heart rate (HR) associated with local anesthesia containing adrenaline [1], bradycardia caused by vasovagal reflexes [2], episodes of hyperventilation, and even apnea due to choking or foreign‑body airway obstruction [3]. These incidental changes may develop into medical emergencies if not recognized promptly. Moreover, they can occur not only in medically compromised patients, but also in younger, otherwise healthy patients [1, 2, 4]. Consequently, monitoring vital signs, such as the respiratory rate (RR) and HR, is important for ensuring patient safety during dental treatment [5].

However, baseline assessment and systematic monitoring of vital signs are rarely performed in routine dental practice [3, 6]. This lack of routine vital signs monitoring may compromise early diagnosis and intervention, thereby potentially increasing the risk of adverse events [5]. Vasovagal syncope is one of the most common medical emergencies encountered during dental treatment and may be preceded by changes in heart rate and other vital signs. When continuous monitoring is not performed, these changes may not be detected before loss of consciousness. Although pulse oximetry is a simple method for monitoring pulse rate, it is not routinely applied. A system that enables the continuous monitoring of vital signs on a dental chair without direct patient contact can facilitate broader implementation and, importantly, enhance clinical safety.

Piezoelectric sensors capable of detecting small mechanical oscillations, such as thoracoabdominal movements and peripheral pulse-related vibrations, have been recently applied using relatively low-cost piezoelectric films. These sensors can extract respiratory and cardiac signals from subtle body vibrations, providing a potential solution for noninvasive vital sign monitoring [7]. However, many conventional piezoelectric sensors are rigid and unsuitable as add-on devices in dental chairs. The Taido Sensor (Sumitomo Riko Company Limited, Nagoya, Japan) is made of a thin and flexible piezoelectric rubber that fits well to various dental chair surfaces. Unlike conventional rigid sensors, its flexibility enables the sensor to maintain close contact with the subject’s body when placed on the contoured surface of a dental chair.

Although the RR and HR can be measured using 1-min recordings, shorter monitoring intervals may be necessary to detect rapid physiological changes. In addition, dental treatment often involves vibrations from the cutting instruments [8, 9]. Although these vibration frequencies are generally outside the physiological range, they may introduce signal distortion and affect signal quality [10]. We hypothesized that a thin, flexible piezoelectric sensor fitted on a dental chair could provide accurate RR and HR measurements over short monitoring intervals despite vibrations generated by dental instruments. The null hypothesis was that the Taido Sensor would not demonstrate agreement with the reference methods. The purpose of this study was to evaluate the accuracy and agreement of the Taido Sensor for continuous monitoring of RR and HR over short analytical intervals compared with reference standard devices and assess its signal resilience under simulated dental vibration conditions.

## Methods

### Study design and participants

This prospective, observational study recruited healthy volunteers aged 20–65 years between April 11, 2023, and May 30, 2023. A total of 15 healthy volunteers (5 males and 10 females; mean age, 35.7 years; range, 25–63 years) participated in this study. The exclusion criteria were as follows: (i) symptomatic respiratory disease; (ii) facial deformities; (iii) hypertension (defined as systolic blood pressure ≥ 140 mmHg or diastolic blood pressure ≥ 100 mmHg or being on antihypertensive medication); (iv) arrhythmia; (v) tachycardia (HR ≥ 100 beats/min); (vi) orthopedic disabilities that interfere with ascending and descending stairs; (vii) serious systemic diseases (e.g., hepatic, renal, cerebrovascular, cardiovascular, or endocrine disorders); (viii) mental disorders; (ix) pregnancy; (x) obesity (body mass index over 30 kg/m^2^); (xi) tooth movement or large dental caries in the anterior maxillary tooth; and (xii) allergy to resin.

### Measurement and variables

**Fig. 1:**
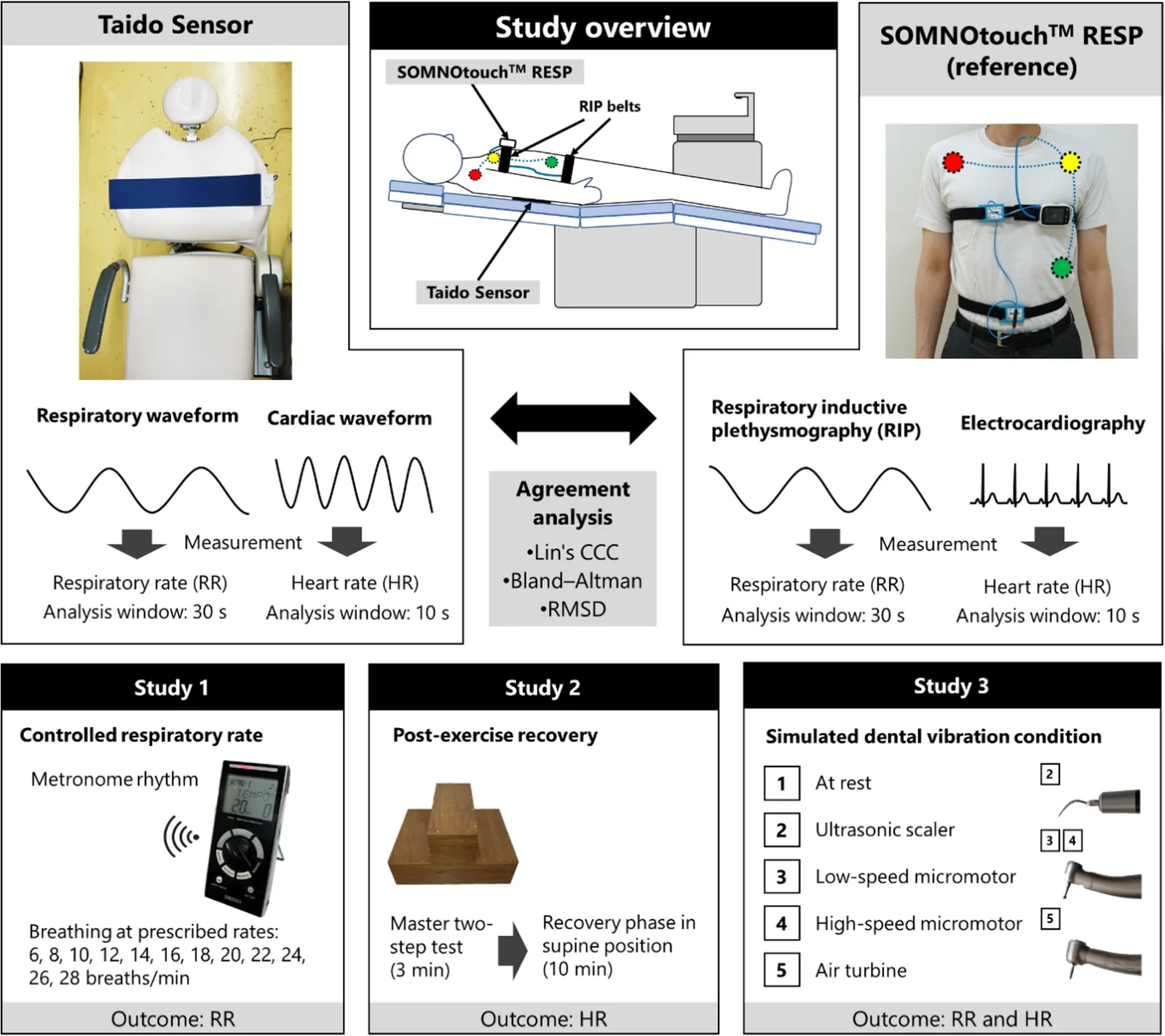
Overview of the study design

RR and HR were measured using two devices: Taido Sensor and SOMNOtouch™ RESP. The study overview, experimental setup, and measurement workflow are shown in Fig. 1.

The Taido Sensor, a flexible piezoelectric rubber sensor, was placed on the surface of the dental chair beneath the thoracic region of the subject to detect subtle body vibrations associated with respiratory and cardiac activity without direct skin contact. Respiratory inductive plethysmography (RIP) belts were placed around the chest and abdomen, and electrocardiography (ECG) was simultaneously recorded using SOMNOtouch™ RESP as the reference standard. The combined RIP waveform was used as the reference respiratory signal. The ECG electrodes were attached directly to the skin beneath the clothing, and dotted lines indicate cables running under the clothing.

Three studies were conducted. In Study 1, subjects performed controlled breathing at prescribed respiratory rates (6, 8, 10, 12, 14, 16, 18, 20, 22, 24, 26, and 28 breaths/min) synchronized with a metronome, and respiratory rate was evaluated. In Study 2, subjects underwent a three-minute Master two-step exercise test, followed by a ten-minute recovery period in the supine position, during which heart rate was assessed. In Study 3, simulated dental vibration conditions were reproduced using an ultrasonic scaler, low-speed micromotor, high-speed micromotor, and air turbine to generate standardized mechanical vibrations.

Agreement between the Taido Sensor and reference measurements was evaluated using Lin’s concordance correlation coefficient (CCC), Bland–Altman analysis, and root mean square deviation (RMSD).

#### Taido sensor

The Taido Sensor is made of a flexible thin piezoelectric rubber. It converts the subtle mechanical vibrations of the body into electrical signals, enabling the acquisition of raw signals containing respiratory, cardiac, and body motion components even when the subject is fully clothed. In this study, the sensor was placed on the surface of a dental chair (Fig. 1). The raw signals were processed to derive respiratory and cardiac waveforms for subsequent analysis. To obtain the respiratory waveform, the raw signal was first bandpass filtered to suppress baseline wander and high-frequency noise and then smoothed using an integration-based procedure to emphasize slow thoracoabdominal movements. For HR estimation, the same signal was processed using a dedicated combination of high- and low-pass filters to highlight the ballistocardiographic activity within the physiological HR range. A detailed implementation of the proprietary filtering algorithms, including specific filter characteristics, is provided by the manufacturer.

#### SOMNOtouch™ RESP

SOMNOtouch™ RESP (FUKUDA DENSHI, Tokyo, Japan) is a diagnostic device for sleep apnea evaluation that records respiratory inductance plethysmography (RIP) and electrocardiogram (ECG) signals. In this study, RIP belts were placed over a thin layer of clothing on both the thorax and abdomen (Fig. 1), and the combined waveform was used as the respiratory signal. ECG signals were recorded using surface electrodes attached directly to the skin at the right shoulder, left shoulder, and left lower thoracic region. This device provides reliable recordings of thoracoabdominal RIP and ECG signals but requires direct attachment to the patient. RR derived from RIP and HR derived from ECG were used as the reference standard for physiological measurements.

#### RR and HR measurements

**Fig. 2:**
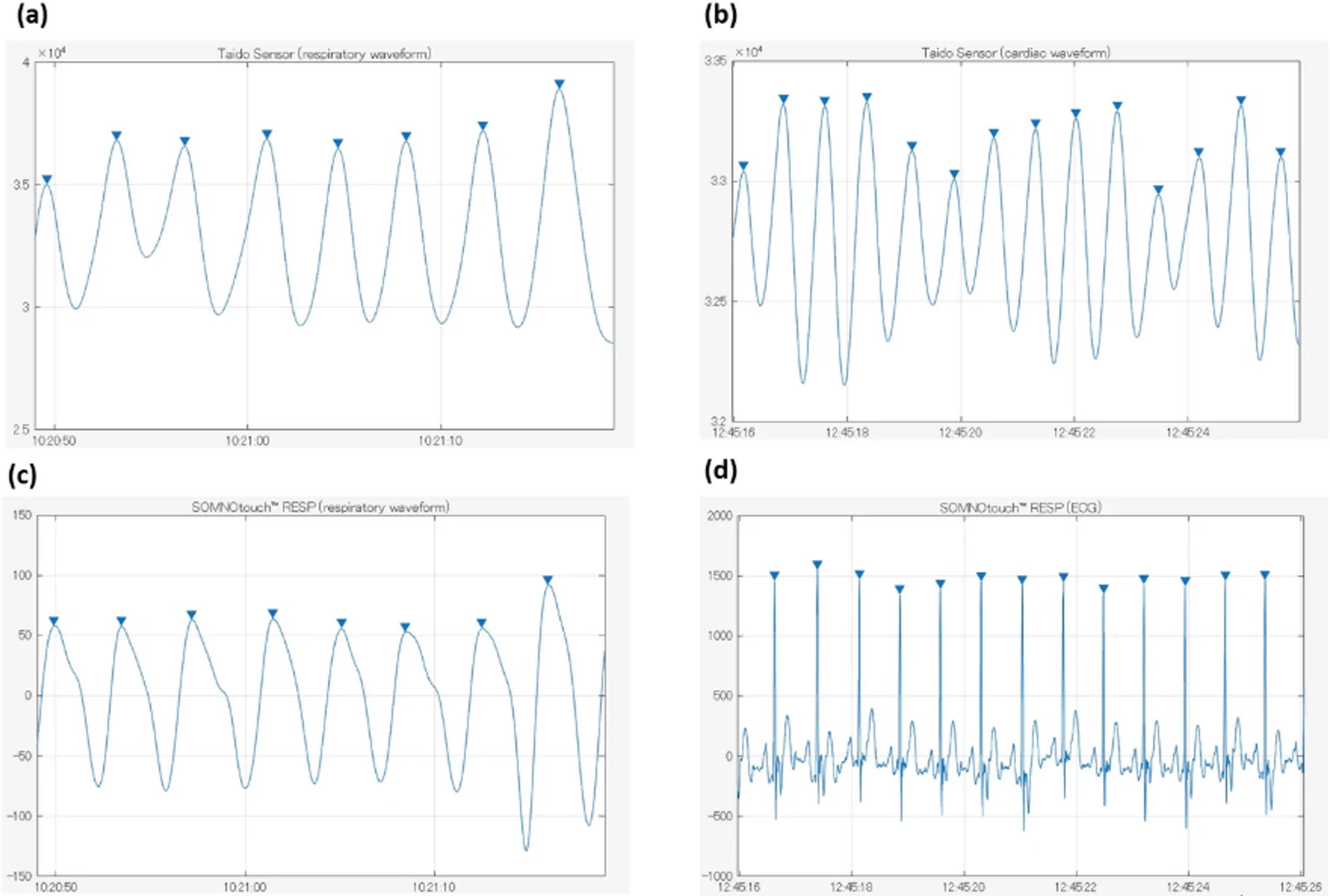
Representative examples of peak detection in reconstructed waveforms from the Taido Sensor and SOMNOtouch RESP. Respiratory and cardiac waveforms were reconstructed using MATLAB, and peaks were identified for inter-peak interval analysis. The upper panels show respiratory (**a**) and cardiac (**b**) waveforms obtained from the Taido Sensor, while the lower panels show the respiratory inductance plethysmography waveform (**c**) and electrocardiogram waveform (**d**) recorded as reference signals using SOMNOtouch RESP

The internal clocks of all measurement devices were synchronized prior to data collection to ensure accurate temporal alignment of waveform data. Respiratory and cardiac waveforms from the Taido Sensor and ECG and RIP waveforms from SOMNOtouch™ RESP were extracted and reconstructed using MATLAB (version R2020a; MathWorks, Inc., Natick, MA, USA). Peaks were detected in all waveforms, and inter-peak intervals were used to derive the RR and HR (Fig. 2). RR was derived from the mean inter-peak interval over a 30-s analysis window using respiratory waveforms recorded by the Taido Sensor and the RIP channel of SOMNOtouch™ RESP. HR was derived from the mean inter-peak interval over a 10-s analysis window using pulse waveforms recorded by the Taido Sensor and R waves from the ECG recorded by SOMNOtouch™ RESP. The sampling frequencies of the Taido Sensor and RIP waveform and ECG of the SOMNOtouch™ RESP were 100, 32, and 256 Hz, respectively.

RR measured by the Taido Sensor (RR-PES) was compared with RR derived from the RIP signal of SOMNOtouch™ RESP (RR-RIP). HR measured by the Taido Sensor (HR-PES) was compared with HR derived from the ECG signal of SOMNOtouch™ RESP (HR-ECG).

### Outcome variables

The primary outcome was the agreement in RR measurements between RR-PES and RR-RIP. The secondary outcome was the agreement in HR measurements between HR-PES and HR-ECG.

### Study conditions

#### Study 1: Controlled RR

In Study 1, the participants wore thoracic and abdominal RIP belts and laid in a supine position on a dental chair fitted with the Taido Sensor. After resting for 3 min, they were instructed to breathe synchronously with a metronome. The RR was sequentially set from 6 to 28 breaths/min in two-step intervals. Each rate was maintained for 2 min while data were recorded.

#### Study 2: Post-exercise HR

The resting HR of the participants was recorded using the ECG signal of SOMNOtouch™ RESP. The participants initially performed a Master’s two-step test for 3 min [11]. Immediately thereafter, they were placed in the supine position on a dental chair fitted with the Taido Sensor, and data were recorded for 10 min. The step-climbing rhythm was decided based on each participant’s age, sex, and weight. If the HR increased to > 160 beats/min or > 3 times the resting HR, the test was terminated.

#### Study 3: Simulated dental vibration conditions

A temporary prosthetic device fabricated using an ultrafast polymerization resin was fitted to the maxillary anterior teeth of the participants (Online Resource 1). After the participants wore the SOMNOtouch™ RESP RIP belts and the ECG electrodes were attached, they were placed in a supine position on a dental chair fitted with the Taido Sensor. Mechanical vibrations were then applied to the temporary prosthetic device using an ultrasonic scaler (Solfy F^®^, Morita, Kyoto, Japan; scaling mode, universal tip), low-speed (250 rev/min) and high-speed (40000 rev/min) micromotors (TORQTECH^®^, Morita, Kyoto, Japan; steel round bur), and an air turbine (TWINPOWER TURBINE 4 H, Morita, Kyoto, Japan; medium mode, diamond bur). Each instrument was directly applied in contact with the resin surface of the prosthetic device. Data were recorded for 1 min in each condition.

### Sample size

Owing to the exploratory nature of this validation study, the lack of prior data for Lin’s concordance correlation coefficient (CCC)-based sample size estimation, and feasibility considerations, the sample size was set at 15.

### Statistical analysis

The agreement in RR and HR measurements between the Taido Sensor and reference methods (RIP and ECG) was assessed using Lin’s CCC [12] and Bland–Altman analysis [13]. Although various statistical approaches are available for validation studies, the combination of CCC and Bland–Altman analysis has been widely used to assess the agreement between sensor-derived and reference measurements [14]. Unlike Pearson’s correlation coefficient, which only measures linear association and fails to detect systematic bias, the CCC evaluates both precision and accuracy by determining how closely the data adhere to the 45-degree line of perfect agreement. In this study, concordance was considered satisfactory if Lin’s CCC was > 0.95. Complementing this, Bland–Altman plots were used to visually and statistically identify the mean difference (fixed bias) and the 95% limits of agreement (mean ± 1.96 standard deviation). Proportional bias was further evaluated using linear regression between the mean and difference values. This approach facilitated the clear clinical interpretation of the agreement between the two methods in routine practice. The root-mean-square deviation (RMSD) was calculated as the average difference between the reference and sensor-derived measurements. All statistical analyses were performed using MATLAB (version R2020a). A p-value of 0.05 was considered significant.

## Results

All 15 participants completed the study (Table 1). Valid data were obtained under all the measurement conditions. The agreement in RR and HR measurements between the two devices is shown in Table 2.

**Table 1:** Subject characteristics

Variable	Value
Age, years	35.7 ± 11.9
Sex, n (male/female)	5/10
Height, cm	162.2 ± 7.7
Weight, kg	53.5 ± 7.4
Body mass index, kg/m^2^	20.2 ± 1.5

Data are presented as the mean ± standard deviation or absolute numbers

**Table 2:** Comparison of respiratory and heart rates measured by the piezoelectric sensor and reference methods

Variable	Study no.	Condition	CCC	RMSD	Fixed Bias (95% CI)	Lower 95% LoA (95% CI)	Upper 95% LoA (95% CI)	*r* value	*p*-value
Respiratory rate	1	Controlled respiratory rate	0.992	0.841	−0.17 (−0.292 to −0.049)	−1.784 (−1.992 to −1.576)	1.443 (1.235 to 1.651)	−0.101	0.181
	3	At rest	0.989	0.654	0.073 (−0.299 to 0.446)	−1.2 (−1.829 to −0.571)	1.347 (0.717 to 1.976)	0.398	0.142
	3	Ultrasonic scaler	0.988	0.61	0.293 (−0.013 to 0.6)	−0.755 (−1.273 to −0.237)	1.341 (0.824 to 1.859)	−0.432	0.108
	3	Low-speed micromotor	0.968	0.85	−0.307 (−0.761 to 0.148)	−1.861 (−2.628 to −1.093)	1.247 (0.48 to 2.015)	0.236	0.396
	3	High-speed micromotor	0.995	0.317	0.1 (−0.073 to 0.273)	−0.49 (−0.782 to −0.199)	0.69 (0.399 to 0.982)	−0.433	0.106
	3	Air turbine	0.997	0.342	−0.153 (−0.328 to 0.022)	−0.752 (−1.047 to −0.456)	0.445 (0.149 to 0.74)	−0.038	0.894
Heart rate	2	Post-exercise heart rate	0.982	3.117	−0.504 (−0.961 to −0.048)	−6.577 (−7.357 to −5.796)	5.568 (4.788 to 6.348)	−0.361	< 0.001
	3	At rest	0.993	1.143	−0.02 (−0.675 to 0.635)	−2.26 (−3.366 to −1.153)	2.22 (1.113 to 3.326)	−0.096	0.733
	3	Ultrasonic scaler	0.997	0.809	−0.127 (−0.585 to 0.331)	−1.692 (−2.466 to −0.919)	1.439 (0.665 to 2.212)	−0.104	0.712
	3	Low-speed micromotor	0.993	1.187	0.453 (−0.176 to 1.082)	−1.697 (−2.76 to −0.635)	2.604 (1.541 to 3.666)	−0.349	0.202
	3	High-speed micromotor	0.954	2.726	0.373 (−0.427 to 1.174)	−4.92 (−7.535 to −2.304)	5.666 (3.051 to 8.281)	−0.229	0.412
	3	Air turbine	0.991	1.38	0.16 (−0.626 to 0.946)	−2.527 (−3.855 to −1.2)	2.847 (1.52 to 4.175)	0.195	0.486

Bias refers to the fixed bias derived from Bland–Altman analysis. The 95% limits of agreement were calculated as bias ± 1.96 × the standard deviation. The 95% confidence intervals are presented for the bias and both the lower and upper LoA. r and p indicate the strength and significance of the proportional bias, respectively, evaluated using linear regression between the mean and difference values in the Bland–Altman plots

*CCC*, Lin’s concordance correlation coefficient; *RMSD*, root mean square deviation; *LoA*, limits of agreement; *CI*, confidence interval

### Study 1: Controlled RR

**Fig. 3:**
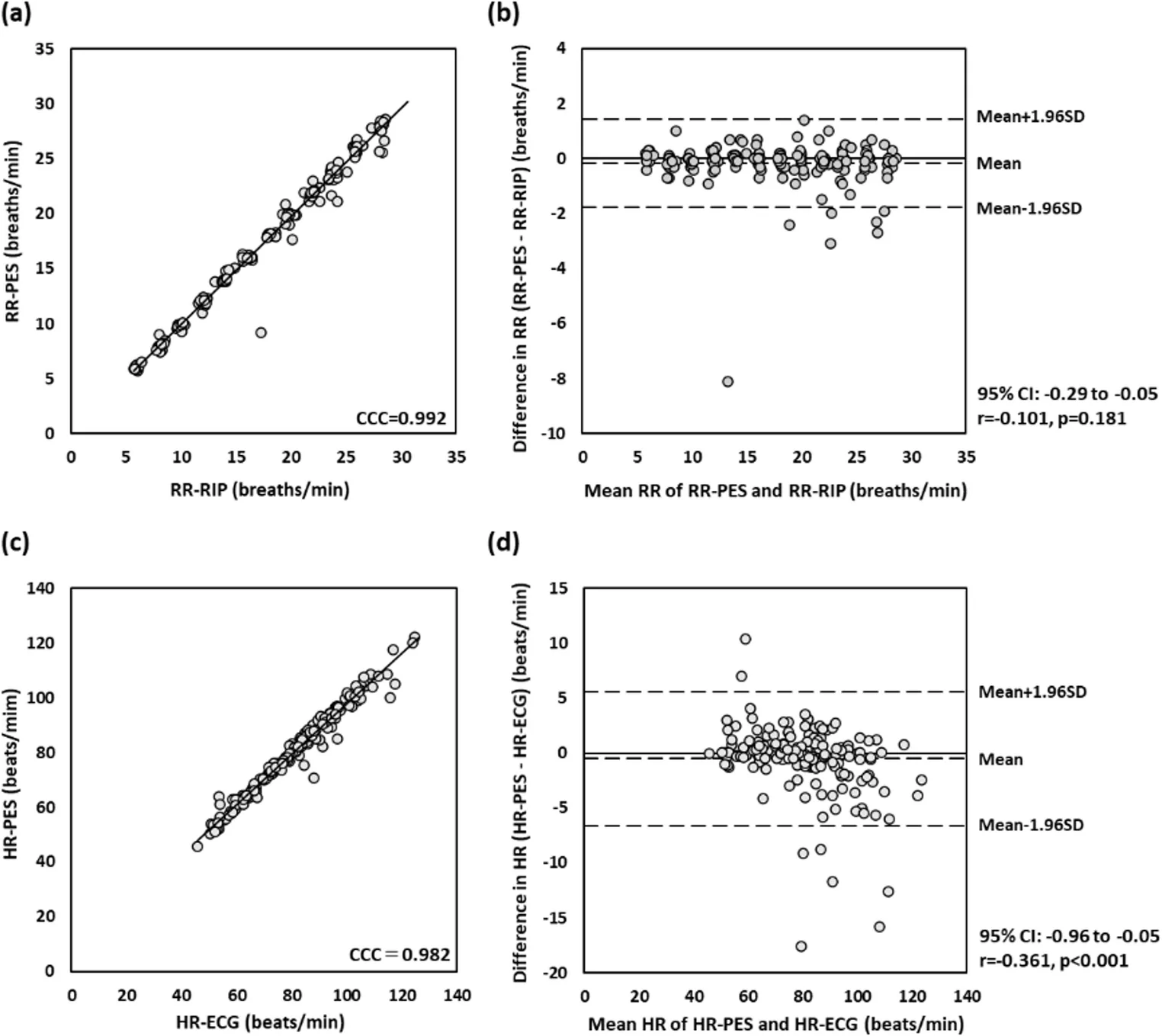
Comparison of respiratory rate (RR) and heart rate (HR) measurements between the Taido Sensor and reference devices. (**a**, **b**) Scatter plot and Bland–Altman plot for RR comparing RR-PES with RR-RIP under controlled breathing conditions (Study 1). (**c**, **d**) Scatter plot and Bland–Altman plot comparing HR-PES with HR-ECG after exercise load (Study 2). The solid lines indicate the line of equality (**a**, **c**) or the line of zero difference (**b**, **d**), and the dotted lines indicate the mean bias and 95% limits of agreement. RR-PES, RR derived from the piezoelectric Taido Sensor; RR-RIP, RR derived from respiratory inductive plethysmography; HR-PES, heart rate derived from the piezoelectric Taido Sensor; HR-ECG, heart rate derived from electrocardiography

RR-PES showed excellent agreement with RR-RIP, with a Lin’s CCC of 0.992. Bland–Altman analysis showed a small fixed bias of − 0.17 breaths/min and narrow limits of agreement of − 1.784 (95% CI: − 1.992 to − 1.576) to 1.443 (95% CI: 1.235 to 1.651). Most measurements were distributed within the limits of agreement across the full range of 6 breaths/min to 28 breaths/min. Although slight underestimation was observed at RRs ≥ 20 breaths/min, overall agreement between the two methods remained high (Fig. 3a, b).

### Study 2: Post-exercise HR

HR-PES showed strong agreement with HR-ECG, with a CCC of 0.982. Bland–Altman analysis showed a fixed bias of − 0.504 beats/min and limits of agreement ranging from − 6.577 beats/min (95% CI: − 7.357 to − 5.796) to 5.568 beats/min (95% CI: 4.788 to 6.348). A significant proportional bias was observed across the measured range (*r* = − 0.361, *p* < 0.001), with greater variability at higher HR values (Fig. 3c, d).

### Study 3: Simulated dental vibration conditions

RR-PES showed high agreement with RR-RIP under the resting condition (CCC = 0.989). The corresponding CCC values during ultrasonic scaler, low-speed micromotor, high-speed micromotor, and air turbine operation were 0.988, 0.968, 0.995, and 0.997, respectively. HR-PES also demonstrated high agreement with HR-ECG under the resting condition (CCC = 0.993). The corresponding CCC values during ultrasonic scaler, low-speed micromotor, high-speed micromotor, and air turbine operation were 0.997, 0.993, 0.954, and 0.991, respectively.

**Fig. 4:**
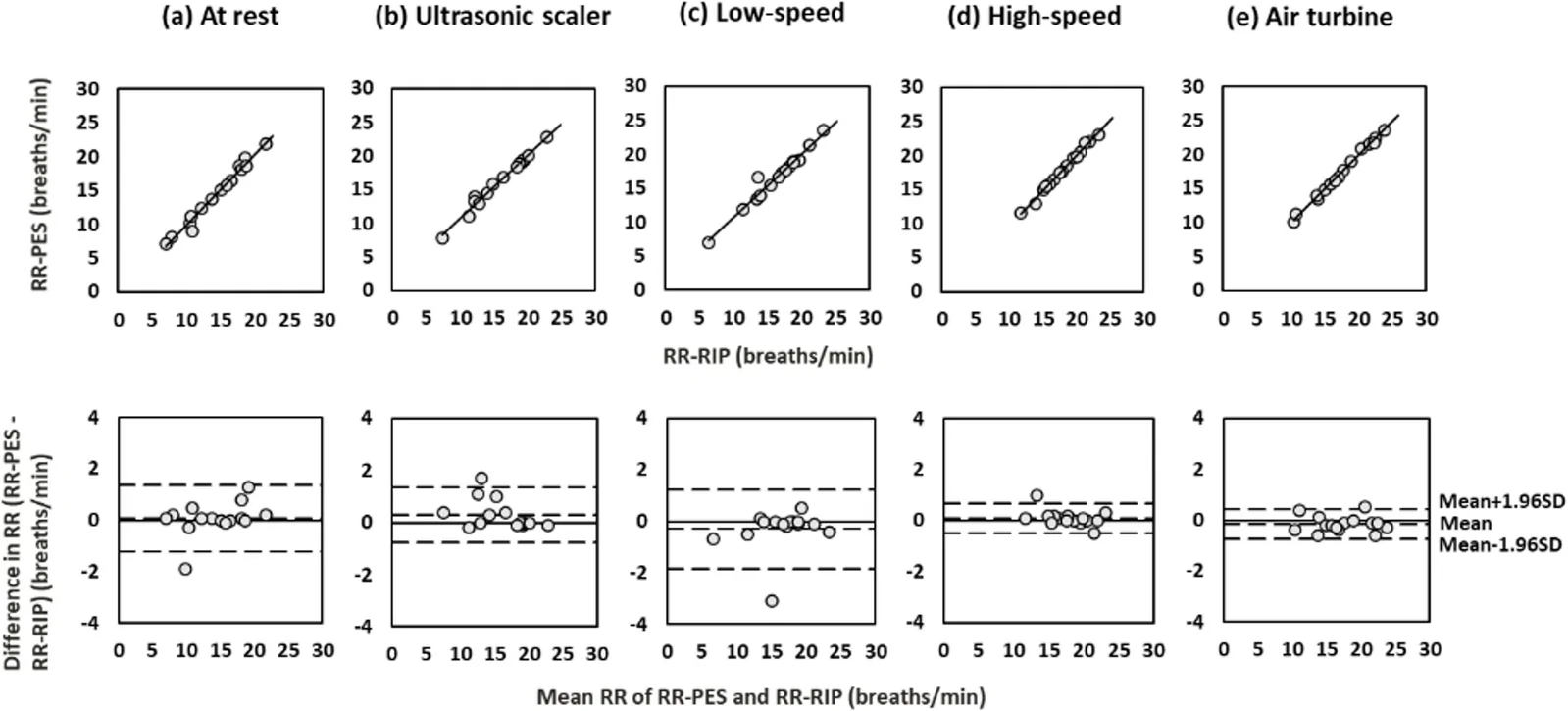
Comparison of respiratory rate measurements between the Taido Sensor and the reference device under various simulated dental treatment conditions. (**a**) At rest, (**b**) ultrasonic scaler, (**c**) low-speed micromotor, (**d**) high-speed micromotor, and (**e**) air turbine

**Fig. 5:**
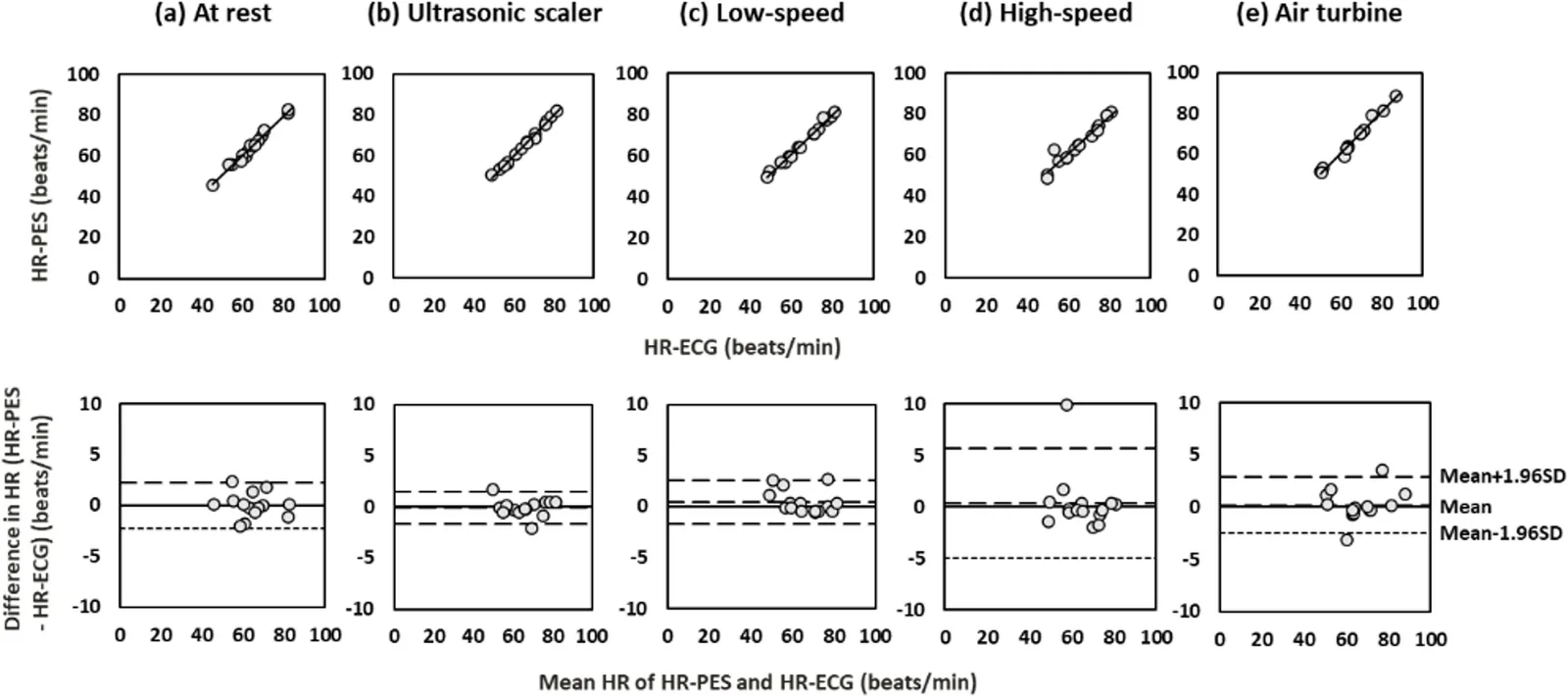
Comparison of heart rate measurements between the Taido Sensor and the reference device under various simulated dental treatment conditions. (**a**) At rest, (**b**) ultrasonic scaler, (**c**) low-speed micromotor, (**d**) high-speed micromotor, (**e**) air turbine. For each condition, the upper panel shows a scatter plot comparing HR-PES with HR-ECG, and the lower panel shows the corresponding Bland–Altman plot. Solid lines indicate the line of equality in the scatter plots and the line of zero difference in the Bland–Altman plots. Dotted lines indicate the mean bias and 95% limits of agreement. HR, heart rate; HR-PES, heart rate derived from the piezoelectric Taido Sensor; HR-ECG, heart rate derived from electrocardiography

Bland–Altman analysis showed mean differences close to zero with acceptable limits of agreement under all conditions. For RR, the p-values for proportional bias were 0.142 at rest and 0.108, 0.396, 0.106, and 0.894 during ultrasonic scaler, low-speed micromotor, high-speed micromotor, and air turbine operation, respectively. For HR, the corresponding p-values were 0.733, 0.712, 0.202, 0.412, and 0.486. No significant proportional bias was observed under any simulated dental vibration condition (Table 2; Figs. 4 and 5).

For each condition, the upper panel shows a scatter plot comparing RR-PES with RR-RIP, and the lower panel shows the corresponding Bland–Altman plot. Solid lines indicate the line of equality in the scatter plots and the line of zero difference in the Bland–Altman plots. Dotted lines indicate the mean bias and 95% limits of agreement. RR, respiratory rate; RR-PES, respiratory rate derived from the piezoelectric Taido Sensor; RR-RIP, respiratory rate derived from respiratory inductive plethysmography.

## Discussion

### Main findings

This study evaluated the accuracy of the Taido Sensor for measuring RR and HR over short time intervals and device resilience against vibrations induced by dental instruments. Our results demonstrated that the Taido Sensor fitted on a dental chair accurately measured RR and HR without requiring direct patient contact. Excellent agreement with the standard reference methods (RIP and ECG) was observed, with Lin’s CCC exceeding 0.95 across all tested conditions. The sensor showed consistent accuracy during controlled breathing, post-exercise recovery, and under vibrations from ultrasonic scalers and rotary dental instruments. Furthermore, reliable estimates were obtained using short-waveform windows (30 s for RR and 10 s for HR), indicating the feasibility of real-time non-contact monitoring during dental procedures.

### RR monitoring

The observed CCC values for RR exceeded 0.99 across the full range of 6 breaths/min to 28 breaths/min. Bland–Altman analysis revealed minimal fixed bias and narrow limits of agreement. The RR measurements derived from the Taido Sensor showed excellent agreement with the reference RIP method across all tested conditions, including those with mechanical vibrations. The CCC values exceeded 0.98, and the RMSD values were low. The differences between methods were within ± 3 breaths/min, a threshold frequently cited as the clinically acceptable limit for agreement between respiratory monitoring devices and reference standards [15, 16]. The findings are also consistent with previous reports describing the interpretation of Bland–Altman analysis and agreement assessment [14]. The narrow distribution of errors observed in this study supports the clinical applicability of the Taido Sensor, even under vibratory dental conditions.

Varying time windows for RR measurements have been used in previous studies. Longer time windows of approximately 5 min have been used in some analytical approaches for RR estimation [17]. However, in clinical settings, 60-s measurements are more commonly regarded as the clinical reference standard for detecting abnormal rates [18, 19]. Very short windows of 15 s have been shown to introduce significant measurement errors [20, 21]. Therefore, the optimal duration of measurement depends on the clinical context. In dental settings, where the rapid detection of respiratory alterations during procedures is essential, intermediate durations (e.g., 30 s) [7] may provide a balance between temporal responsiveness and measurement reliability.

### HR monitoring

Consistent with the findings for RR measurements, HR measurements derived from the Taido Sensor showed excellent agreement with ECG-based reference values across all conditions. Lin’s CCC exceeded 0.95 in all settings, including post-exercise recovery and various simulated dental vibrations, confirming clinically acceptable agreement. Although the Bland-Altman analysis indicated slightly greater variability at higher HR values, the overall accuracy under typical physiological conditions was maintained. These findings are consistent with previous reports on contactless piezoelectric sensors that have demonstrated strong agreement with ECG in hospital and clinical settings [22, 23].

In non-contact monitoring using ballistocardiography (BCG), signals are weak and susceptible to motion artifacts, making reliable beat-to-beat estimation difficult [24]. Although a 1‑min window is often used as a clinical reference under stable conditions, dental procedures often involve intermittent patient motion and unavoidable vibrations from cutting instruments. In BCG monitoring, shorter analysis windows improve temporal resolution, whereas longer windows improve signal stability. Very short windows of approximately 5 s improve the responsiveness; however, they are highly sensitive to noise. In contrast, although longer windows of 30–60 s may enhance stability, they may also delay the detection of acute tachycardia or bradycardia [25]. An intermediate window of 10 s provides a practical compromise, enabling sufficient noise reduction while preserving the clinically relevant temporal variations necessary for real-time safety monitoring [26]. The slight bias observed at higher HR may be attributed to the reduced signal amplitude and shortened interbeat intervals [23]. Although improvements in the detection algorithm may be required at higher HR values, the current accuracy appears to be sufficient for monitoring physiological changes during dental treatment.

### Signal robustness under mechanical interference

Both air-turbine handpieces and micromotor-driven devices inevitably introduce high-frequency mechanical vibrations into the clinical environment during routine procedures. These vibrations are predominantly high-frequency signals that exceed the frequency ranges of physiological respiration and cardiac activity [27]. In addition, they are transmitted via the handpiece to the connected structures, instead of being confined to the treatment site [8, 28]. Such vibratory stimuli are perceptible to patients and contribute to treatment-related discomfort [29]. These findings suggest that mechanical disturbances are both clinically relevant and unavoidable.

The Taido Sensor maintained stable signal quality even during the use of ultrasonic scalers, micromotors, and air turbines, likely reflecting the flexibility of its piezoelectric material and signal processing methods designed to reduce vibration-related noise. In contrast, BCG-based monitoring can be affected by motion artifacts and environmental noise [30]. Given that clinical environments introduce high-frequency noise that may fold into lower-frequency components, signal distortion cannot be entirely ruled out [10]. Accordingly, even when dental vibration frequencies are outside the physiological range, their impact on signal quality should be considered. The present findings indicate that the Taido Sensor enables stable signal acquisition under mechanically complex conditions, thereby supporting its applicability in real-world dental settings.

### Clinical implications

The present study demonstrates that the Taido Sensor enables non-invasive and clinically reliable monitoring of RR and HR with sufficient accuracy, even at short analysis intervals. Continuous assessment of both the RR and HR without direct skin contact simplifies setup, reduces patient burden, and enhances the clinical feasibility of routine monitoring. The system also appeared relatively robust against vibration-related interference under the tested conditions, which is particularly advantageous in the dental environment. Such non-invasive continuous monitoring is expected to facilitate the early detection of unexpected physiological events, including tachycardia, bradycardia, hyperventilation, and airway obstruction, addressing the need for continuous monitoring for earlier recognition of patient deterioration [31].

Although contact-free and wearable monitoring systems have been increasingly investigated in sleep medicine and hospitalized patients [31, 32], their application in routine dental practice remains limited. The Taido Sensor can be integrated into newly designed dental chairs or retrofitted for existing units to facilitate its use in routine clinical practice. Although not intended to replace standard monitoring in high-risk patients, the sensor provides a practical and clinically meaningful alternative in settings where continuous monitoring is not routinely performed.

### Limitations

This study has some limitations. First, the study was conducted in a simulated environment using healthy volunteers. Further studies are needed in actual clinical settings, where patient movement and operator-related factors may affect signal acquisition. Second, the effects of conditions that may interfere with accurate signal acquisition, such as arrhythmia, obstructive respiratory disease, and musculoskeletal abnormalities, were not evaluated. Third, the effects of facial or jaw movement, swallowing, and suctioning on signal quality were also not evaluated. Fourth, the accuracy of the HR estimation decreased at values above 90 beats/min, indicating the need for further refinement of the detection algorithms for higher HR ranges. The generalizability of our findings to routine dental practice should be interpreted with caution. In addition, although repeated measurements were obtained from the same subjects under different physiological conditions, statistical analyses were performed without accounting for within-subject correlation. This approach assumes independence among measurement pairs and may therefore affect the estimation of agreement and precision. Although this limitation is common in validation studies, future analyses using a mixed-effects model may provide more robust estimates, particularly for repeated-measures designs.

However, the enrollment of healthy volunteers under standardized experimental conditions also has several advantages. This design allowed for the evaluation of sensor performance under controlled conditions that reflected important aspects of the dental environment, including patient posture, breathing patterns, exercise-induced tachycardia, and vibrations generated by commonly used dental instruments. It is difficult to reproduce these factors consistently in actual patients. Therefore, although the present findings did not establish the clinical applicability of this technology during routine dental treatments, they provide preliminary evidence of its feasibility and may serve as a basis for future clinical studies.

## Conclusion

The Taido Sensor can accurately monitor RR and HR under simulated dental treatment conditions, including mechanical interference. These findings support the potential integration of flexible piezoelectric sensors into dental chairs for routine patient monitoring.

## Supplementary Information

Below is the link to the electronic supplementary material.

Supplementary File 1 (DOCX 454 KB)

## Data Availability

Data are available from the corresponding author on reasonable request.
