# Supplementary material for: A flexible piezoelectric sensor for non-contact monitoring of respiratory and heart rates: accuracy and clinical feasibility under simulated dental treatment conditions
Source: Clin Oral Investig. 2026 Sep 26;30(10):478. doi: 10.1007/s00784-026-07161-7 (PMC13615905; doi:10.1007/s00784-026-07161-7)
Supplement: Supplementary file 1 [file 784_2026_7161_MOESM1_ESM.docx]

Online Resource 1

Photograph of the temporary resin prosthetic device fitted to the maxillary anterior teeth for simulated dental vibration conditions in Study 3. An ultrasonic scaler, low-speed micromotor, high-speed micromotor, and air-turbine handpieces were used to deliver standardized mechanical vibrations.
